# Children’s sex composition and modern contraceptive use among mothers in Bangladesh

**DOI:** 10.1371/journal.pone.0297658

**Published:** 2024-05-31

**Authors:** Md. Nuruzzaman Khan, Shimlin Jahan Khanam, Md Arif Billah, Md Mostaured Ali Khan, M Mofizul Islam

**Affiliations:** 1 Department of Population Science, Jatiya Kabi Kazi Nazrul Islam University, Mymensingh, Bangladesh; 2 Faculty of Business, Economic and Social Development, Universiti Malaysia Terengganu, Kuala Terengganu, Malaysia; 3 Maternal and Child Health Division, icddr,b, Mohakhali, Dhaka, Bangladesh; 4 Department of Public Health, La Trobe University, Melbourne, Australia; University of Salamanca, SPAIN

## Abstract

**Background:**

The stagnation and relatively low use of modern contraceptives are ongoing public health concerns in Bangladesh and other low- and middle-income countries. Although a cultural preference for sons may be linked to the current use of contraceptives, this linkage has not been adequately explored in the Bangladesh context. We investigated the effects of child sex composition on the current use of modern contraceptives.

**Methods:**

We extracted and analysed data from 17,333 women who participated in the 2017/18 Bangladesh Demographic and Health Survey. The outcome variable was the current use of modern contraceptive methods. The study factor was the parity and sex composition of the living children. We used multilevel logistic regressions to determine the association between the study factor and outcome variables, adjusting for potential covariates at the individual-, household-, and community-levels.

**Results:**

Women with relatively high parity had higher odds of currently using modern contraceptives. Among the individual parities, compared to women with no live sons, women with one or more live sons were more likely to report currently using modern contraceptives. However, this association is significant for women up to three children. When examining both parity and children’s sex composition in a regression model, in each parity category, the likelihood of using modern contraceptives tend to rise with an increasing number of sons compared to women with just one daughter.

**Conclusion:**

The findings of this study suggest that while the use of modern contraceptives by women increases with the increasing number of children and son preference is prevalent in Bangladesh, women also want to have a mixed composition of son and daughter. The study findings can be used in family planning programmes to customise contraceptive promotion and counselling messages.

## Introduction

Within just fifty years of independence, Bangladesh achieved remarkable success in improving maternal and child health compared to many other low- and middle-income countries (LMICs) [1]. Meeting the under-five mortality reduction target (38 deaths per 1000 live births) and getting close to the maternal mortality reduction target (196 maternal deaths per 100,000 live births) were the major indicators of its success during the Millennium Development Goals (MDGs) period of 2000 to 2015 [1,2]. Bangladesh stood out as one of the few LMICs to meet these targets, while the overall LMIC figure remained at 43 under-five deaths per 1000 live births and 220 maternal deaths per 100,000 live births [4]. This success has been attributed to the country’s consistent emphasis on maternal and child health improvement since its independence in 1971 through the implementation of several two- and five-year plans as well as health, nutrition, and population strategies [3]. For example, Bangladesh’s success in ensuring antenatal healthcare services and contraceptive use as well as reducing the unmet need for contraceptives, which were also targets of the MDGs, contributed significantly to these achievements [1,3].

However, since the end of 2015, when the MDGs concluded and the Sustainable Development Goals (SDGs) were introduced with more ambitious targets for reducing maternal and child mortality by 2030, Bangladesh has experienced a stagnation in its progress towards reducing maternal and child mortality [4–6]. This stagnation, along with while an increase in the number of women of reproductive age, indicates that more women have unmet needs for contraception [7,8]. Consequently, more women are facing unintended pregnancies, although the rate has remained constant at 47 per 100 conceptions, compared to the estimate of 49 per 100 conceptions for LMICs [7]. However, following live births, this figure goes down to 23 per 100 live births, indicating a significant rate of abortion, though it is prohibited by law in Bangladesh [3,7]. Post-abortion complications contribute to around 13% of overall maternal mortality. Women who continue to have unintended pregnancies are also less likely to use maternal healthcare services, which increases adverse maternal and child health outcomes, including pregnancy complications, stillbirth, and maternal and child mortality.

The decision to use contraception is influenced by a complex interplay of factors involving women and their partners, including individual-level factors (such as women’s age, education, and employment) and household-level factors (such as partner’s education, occupation, number of children ever born, and wealth quintile) [9–18]. Community- (such as place of residence and region) and healthcare facility-level factors (such as the availability of contraceptives at the healthcare facility) and cultural factors can also influence this decision [9–19]. For example, in societies with a strong cultural preference for male children, couples with only daughters are less likely to use contraception than those with at least one son [20–24].

Historically, like other LMICs, son preference is widespread in Bangladesh; couples with two or more daughters often do not use contraceptives with the hope that the next baby will be a son [19,23,25]. Also, many of those who use contraception rely on traditional methods such as periodic abstinence and withdrawal, which have high failure rates [26]. Reliance on traditional methods may, to some extent, be attributed to inadequate knowledge. However, in recent years, there has been significant progress in women’s education and empowerment, which may have potentially broken this long-standing cultural norm. As a result, children’s sex composition may no longer have a significant influence on contraceptive use [19,27]. However, there is inadequate data on this from Bangladesh and other LMICs, although it is critical to have up-to-date information [19,22,23,25,28]. Therefore, this study aims to investigate the association between children’s sex composition and women’s current use of modern contraception in the Bangladesh context, adjusted for potential confounders at the individual, household, and community levels.

## Methods

### Survey design and sampling

We analysed Bangladesh Demographic and Health Survey (BDHS) data collected during 2017–18. BDHS is a large-scale, nationally representative and repeat-cross-sectional survey conducted every three years as part of the Demographic and Health Survey program. The National Institute of Population Research and Training conducted this survey. The survey used a multistage stratified random sampling method. In the first stage of sampling, a total of 675 primary sampling units (PSUs) were selected from a list of 293,579, which were generated by the Bangladesh Bureau of Statistics. The selection process ensured the representativeness of each division, district, and rural and urban areas. In the second stage, a fixed number of 30 households from each cluster were chosen using systematic random sampling methods. This resulted in a list of 20,160 households, from which data were collected from 19,457 households (a 96% inclusion rate). There were 20,376 eligible women aged 15 to 49 who were regular residents of or lived in those households the night before the survey date. Data were collected from 20,127 eligible women, with a response rate of 98.8%. Details about the sampling procedure, sample size, design, and sample weights in BDHS have been published elsewhere [4].

### Sample size of this study

The study sample was restricted to mothers who were sexually active, and not pregnant at the time of the survey. A total of 17,333 mothers, which accounted for 86.61% of the total sample, met these criteria and were thus chosen for analysis. The remaining 13.9% were excluded because they were pregnant or had no children at the time of the survey.

### Outcome variables

The outcome variable was the current use of modern contraception. To determine women’s current contraceptive usage status, they were first asked, "*Are you or your husband currently doing something or using any method to delay or avoid getting pregnant*?" Responses were recorded dichotomously as "1 = Yes" or "0 = No". Women who responded affirmatively were then asked, "*Which method are you using*?" If women reported multiple contraceptive methods, the most effective method used was recorded. Using these responses, we generated a dichotomously coded variable where women who reported using any modern contraceptives were coded "1 = Yes" and those who reported no use or using only traditional contraceptive methods were coded "0 = No".

### Explanatory variables

The key independent variables of interest were the number of living children and their sex composition. We first generated a continuous variable counting the number of living children of individual women at the time of the survey. We then categorised this variable depending on children’s sex composition under different parities. To examine the effects of both parity and sex composition, we also created a categorical variable for women up to four children. The categories are mutually exclusive and start with no son & one daughter and continue up to four sons & no daughters.

### Covariates

We conducted a three-stage process to select covariates for our study. Initially, we compiled a list of covariates based on their demonstrated associations with the current use of modern contraception, as noted in several previous studies in LMICs and Bangladesh [19,20,22,25,28]. Subsequently, we verified the availability of these selected variables in the survey dataset to ensure their inclusion. Finally, we assessed the statistical significance of the available variables related to the current use of modern contraception by employing the chi-square test on the analysed survey data (S1 Table). Only the covariates found to be statistically significant were included in our analysis. The covariates included in the analysis were characteristics of women, children, households and communities. Children’s characteristics included whether any children died (none, at least one). Mothers’ characteristics were their age (15–19, 20–34, ≥35 years), education levels (no formal education, primary, secondary, higher), and current formal employment status (unemployed, employed). Household characteristics were husbands’ education (no formal education, primary, secondary, higher), husbands’ occupation (agricultural worker, physical worker, business, service), wealth index (poorest, poorer, middle, richer, richest), religion (Islam, others) and exposure to mass media (low, moderate, high). Community-level characteristics were the place of residence (rural, urban) and regions (Barishal, Chattogram, Dhaka, Khulna, Mymensingh, Rajshahi, Rangpur and Sylhet).

### Statistical analysis

The characteristics of the respondents, their children, households, and communities were described using frequency and percentage. In the BDHS survey, individual women were nested within households and households were nested within clusters/primary sampling units. To account for this multiple hierarchy and dependency in the data, we performed multilevel logistic regressions and explored the associations between the study factor and the current use of modern contraception while adjusting for potential confounders. We additionally compared the results generated from the multilevel logistic regression model (random-effects model) with the simple logistic regression model (fixed effect model) through the likelihood ratio test. To conduct this comparison, we employed the same list of variables for both the random-effects and fixed-effect models. The test found statistically significant results, indicating multilevel mixed-effect regression is preferable for modelling this data. Two-level multilevel regressions were conducted: children (level 1) and community (level 2). Primary sampling units were considered communities. We conducted regressions to estimate the association for the total number of children a woman gave birth to and then separate regressions for parity 1, parity 2, parity 3 and parity 4. The results were reported as the adjusted odds ratio (aOR) with their corresponding 95% confidence interval (95% CI). All the analyses were done considering the complex survey design and weights of the sample. Statistical software Stata version 15.2 was used for all analyses.

### Ethics approval

Prior to conducting the survey in Bangladesh, ethical approval was obtained from the institutional review board of the ICF, USA, and subsequently from the National Research Ethics Committee of the Bangladesh Medical Research Council. Informed written consent was obtained from all individuals involved, utilising an appropriate institutional form. In our research, we sought permission to access the data for analytical purposes, and the survey authority provided us with non-identified data. As the study involved secondary data analysis and adhered to the relevant guidelines and regulations, no additional ethical approval was required.

## Results

### Background characteristics

Background characteristics of the respondents, their children, households, and communities are presented in Table 1. Almost 32% of women had two children. At the time of the survey, over half of the women were 20–34 years old and 32.8% received up to primary and 38.4% received up to secondary education. Around 32% of women reported that their husbands had completed only the primary level of education. The most common occupation of women’s husbands was labourer (45.5%), followed by agriculture work (27.9%) and business (21.4%). Approximately 56% of all respondents reported having moderate exposure to mass media.

**Table 1:** Background characteristics of the respondents.

Characteristics	Distribution among all eligible women (n = 17333)		Among women currently using modern contraceptives (n = 9439)	
	n	%	n	%
**Children characteristics**				
**Parity**				
1	3844	22.2	1956	20.7
2	5495	31.7	3327	35.3
3	3859	22.3	2222	23.5
4	2152	12.4	1072	11.4
5 or more	1982	11.4	861	9.1
**History of the death of a child women gave birth to**				
None	14425	83.2	8077	85.6
At least one	2908	16.8	1362	14.4
**Sex composition of the existing children**				
No son	3654	21.1	1882	19.9
At least one son	13678	78.9	7564	80.1
***Mother’s characteristics***				
**Age-group**				
15–19	964	5.6	607	6.4
20–34	9094	52.5	5640	59.8
≥35	7275	42.0	3192	33.8
**Education level**				
No formal education	3154	18.2	1420	15.1
Primary	5690	32.8	3118	33.4
Secondary	6648	38.4	3833	40.6
Higher	1840	10.6	1067	11.3
**Respondent’s employment status**				
Unemployed	8626	49.8	4473	47.4
Employed	8706	50.2	4965	52.6
**Religion**				
Muslim	15659	90.3	8414	89.1
Others	1674	9.7	1025	10.9
**Exposure to mass media**				
Low	6019	34.7	3120	33.1
Moderate	9787	56.5	5447	57.7
High	1526	8.8	872	9.2
***Household’s characteristics***				
**Husband’s education**				
No formal education	3786	23.2	2179	23.1
Primary	5313	32.6	3200	34.0
Secondary	4738	29.1	2643	28.0
Higher	2470	15.1	1406	14.9
**Husband’s occupation**				
Agriculture	4443	27.9	2706	29.1
Labourer	7249	45.5	3927	42.2
Services	820	5.2	485	5.2
Business	3413	21.4	2183	23.5
**Wealth index**				
Poorest	3297	19.0	1941	20.6
Poorer	3443	19.9	1884	20.0
Middle	3530	20.4	1869	19.8
Rich	3533	20.4	1932	20.5
Richest	3529	20.4	1812	19.2
***Community-level characteristics***				
**Place of residence**				
Rural	12450	71.8	2788	29.5
Urban	4882	28.2	6651	70.5
**Region**				
Barishal	983	5.7	512	5.4
Chattogram	3135	18.1	1495	15.8
Dhaka	4304	24.8	2410	25.5
Khulna	2034	11.7	1089	11.5
Mymensingh	1317	7.6	774	8.2
Rajshahi	2445	14.1	1401	14.8
Rangpur	2108	12.2	1283	13.6
Sylhet	1005	5.8	475	5.0

**Note:** All percentages are weighted.

The distribution of modern contraceptive use across the characteristics of the respondents, their children, households, and communities is presented in Table 1. Of the women who reported currently using modern contraceptives, around 35.3% had at least two children. The majority of women who reported using modern contraceptive methods were between the ages of 20 and 34 (59.8%), completed up to secondary education (40.6%) and lived in rural areas (70.5%).

### Women’s current use of modern contraceptives

Table 2 presents the distribution of contraception use. Nearly 55% of the women reported using modern contraception. Pills and injectables are the most dominant forms of modern contraception.

**Table 2:** Pattern of contraceptive use in Bangladesh (n = 17,333).

Type of contraception	n (%)
**Modern methods of contraception**	**9439 (54.5)**
Oral contraceptive pill	4563 (48.3)
Injectable	2034 (21.6)
Condoms	1218 (12.9)
Female sterilization	915 (9.7)
Male sterilization	197 (2.1)
Intra Uterine Device	105 (1.1)
Implants	405 (4.3)
**No use or use of only traditional methods**	**7894 (45.5)**
Periodic abstinence	1289 (16.3)
Withdrawal	475 (6.02)
No use of contraception	6082 (77.1)

Note: All percentages are weighted.

### Association between current use of modern contraceptives and number of living children

Table 3 presents the results of the multilevel logistic regression model examining the association between the number of living children and the use of modern contraception (the complete results of the relevant full model are provided in S2 Table). Compared to mothers with one child, those with 2 children (aOR, 1.83; 95% CI, 1.65–2.04), 3 children (aOR, 2.17; 95% CI, 1.92–2.42), 4 children (aOR, 1.99; 95% CI, 1.71–2.31), and 5 or more children (aOR, 1.91; 95% CI, 1.61–2.27) were more likely to report current use of modern contraception. Of the covariates considered for adjustment, several were found to be significantly associated with women’s use of modern contraception. Women who experienced the death of a child had lower odds of using modern contraceptives (aOR, 0.76; 95% CI, 0.69–0.85). Women who have at least one living son had significantly higher odds of reporting that they were using modern contraceptive methods (aOR, 1.22; 95% CI, 1.12–1.34). Women’s increasing age was found to be associated with decreased odds of using modern contraceptive methods as compared to women aged 15–19 years (Table 3). Women from affluent households had lower odds of currently using modern contraception compared to those from resource-poor households. Women who resided in urban areas (aOR, 1.53; 95% CI, 1.36–1.72), were not Muslims (aOR, 1.34; 95% CI, 1.17–1.53), compared to those living in rural areas and who were Muslims, respectively, had lower odds of currently using modern contraceptives.

**Table 3:** Multilevel logistic regression examining the associations of women’s current use of modern contraceptives with child, mother, household and community characteristics.

Variables	Women currently using modern contraception methods		
Parity	aOR	95% CI	p-value
1 (reference)	1		
2	1.83	1.65–2.04	<0.01
3	2.17	1.92–2.46	<0.01
4	1.99	1.71–2.31	<0.01
5 or more	1.91	1.61–2.27	<0.01
**History of the death of a child women gave birth to**			
None (reference)	1		
At least one	0.76	0.69–0.85	<0.01
**Sex composition of the existing children**			
No son (reference)	1		
At least one son	1.22	1.12–1.34	<0.01
***Mother’s characteristics***			
**Age-group**			
15–19 (reference)	1		
20–34	0.59	0.50–0.69	<0.01
≥35	0.25	0.20–0.29	<0.01
**Education level**			
Not formal education (reference)	1		
Primary	1.23	1.10–1.37	0.39
Secondary	1.40	1.24–1.58	<0.01
Higher	1.66	1.39–1.98	<0.01
**Respondent’s employment status**			
Unemployed (reference)	1		
Employed	1.31	1.21–1.41	<0.01
***Household’s characteristics***			
**Husband’s education**			
No formal education (reference)	1		
Primary	0.96	0.87–1.06	0.39
Secondary	0.79	0.71–0.89	<0.01
Higher	0.76	0.65–0.89	<0.01
**Husband’s occupation**			
Agriculture (reference)	1		
Physical	0.66	0.60–0.72	<0.01
Services	0.99	0.82–1.21	0.94
Business	1.10	0.98–1.22	0.09
**Wealth index**			
Poorest (reference)	1		
Poorer	0.76	0.68–0.85	<0.01
Middle	0.70	0.62–0.80	<0.01
Rich	0.66	0.58–0.76	<0.01
Richest	0.51	0.44–0.60	<0.01
**Religion**			
Muslim (reference)	1		
Others	1.34	1.17–1.53	<0.01
**Exposure to mass media**			
No (reference)	1		
Moderate	1.27	1.16–1.38	<0.01
High	1.36	1.17–1.59	<0.01
***Community-level characteristics***			
**Place of residence**			
Rural (reference)	1		
Urban	1.53	1.36–1.72	<0.01
**Regions**			
Barishal (reference)	1		
Chattogram	0.83	0.68–1.03	0.09
Dhaka	1.20	0.98–1.48	0.08
Khulna	0.98	0.79–1.22	0.88
Mymensingh	1.21	0.96–1.52	0.11
Rajshahi	1.22	0.98–1.51	0.07
Rangpur	1.21	0.97–1.50	0.09
Sylhet	0.86	0.68–1.10	0.24
**Constant**	1.61	1.23–2.11	<0.01
***p* for LR test vs. logistic regression**	185.5		<0.01
**Random effect variance**	1.60	0.13–0.20	-

Note: Survey weight was applied in the regression.

**Fig 1:**
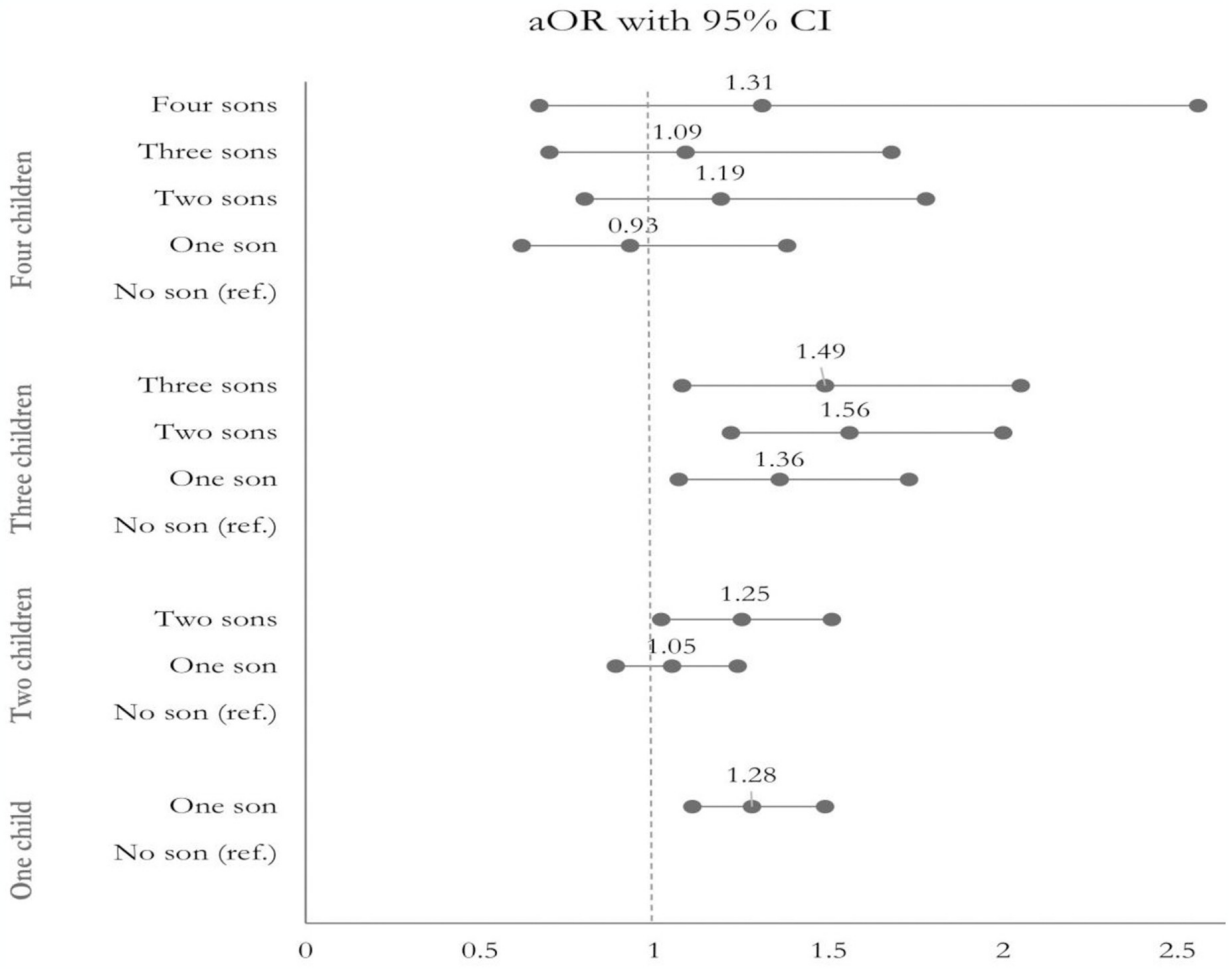
Effects of sex composition on current use of modern contraceptive methods for various parities, controlling for covariates mentioned in Table 3.

Table 4 presents the adjusted association between association between children’s sex composition, their numbers and women’s current use of modern contraception and parities. Overall, the odds of women currently using modern contraceptives were mostly higher if they had one or more male children. Quite opposite associations are also apparent; women who had only daughters had lower odds of currently using modern contraceptives. The associations are insignificant for higher parities (Fig 1). Women with parities 1 and 3 and who had no sons had significantly lower odds of using modern contraceptives. However, the associations were not significant for parities 2 and 3 (Table 4).

**Table 4:** Multilevel regression examining women’s current use of modern contraception by sex composition under different parity of living children adjusted for individual-, household- and community-level characteristics.

Parity	aOR (95% CI)	p-value	Parity	aOR (95% CI)	p-value	Parity	aOR (95% CI)	p-value
**One child**			**One child**			**One child**		
No son (ref.)	1		No daughter (ref.)	1		One son (ref.)	1	
One son	1.28 (1.11–1.49)	<0.01	One daughter	0.78 (0.67–0.90)	<0.01	No son	0.78 (0.67–0.90)	<0.01
**Two children**			**Two children**			**Two children**		
No son (ref.)	1		No daughter (ref.)	1		At least one son (ref.)	1	
One son	1.05 (0.89–1.24)	0.55	One daughter	0.86 (0.74–0.99)	0.04	No son	0.91 (0.77–1.06)	0.23
Two sons	1.25 (1.02–1.51)	0.03	Two daughters	0.81 (0.66–0.98)	0.03			
**Three children**			**Three children**			**Three children**		
No son (ref.)	1		No daughter (ref.)	1		At least one son (ref.)	1	
One son	1.36 (1.07–1.73)	0.01	One daughter	0.93 (0.74–1.17)	0.55	No son	0.69 (0.55–0.86)	<0.01
Two sons	1.56 (1.22–2.00)	<0.01	Two daughters	0.80 (0.63–1.02)	0.07			
Three sons	1.49 (1.08–2.05)	0.02	Three daughters	0.68 (0.49–0.94)	0.02			
**Four children**			**Four children**			**Four children**		
No son (ref.)	1		No daughter (ref.)	1		At least one son (ref.)	1	
One son	0.93 (0.62–1.38)	0.71	One daughter	0.82 (0.55–1.23)	0.34	No son	0.94 (0.64–1.36)	0.73
Two sons	1.19 (0.80–1.78)	0.38	Two daughters	0.87 (0.58–1.29)	0.49			
Three sons	1.09 (0.70–1.68)	0.71	Three daughters	0.78 (0.51–1.21)	0.27			
Four sons	1.31 (0.67–2.56)	0.44	Four daughters	0.89 (0.48–1.64)	0.70			

**Note:** Adjusted for all relevant covariates mentioned in Table 4; ref. means reference group; aOR means adjusted odds ratio; CI means confidence internal.

**Fig 2:**
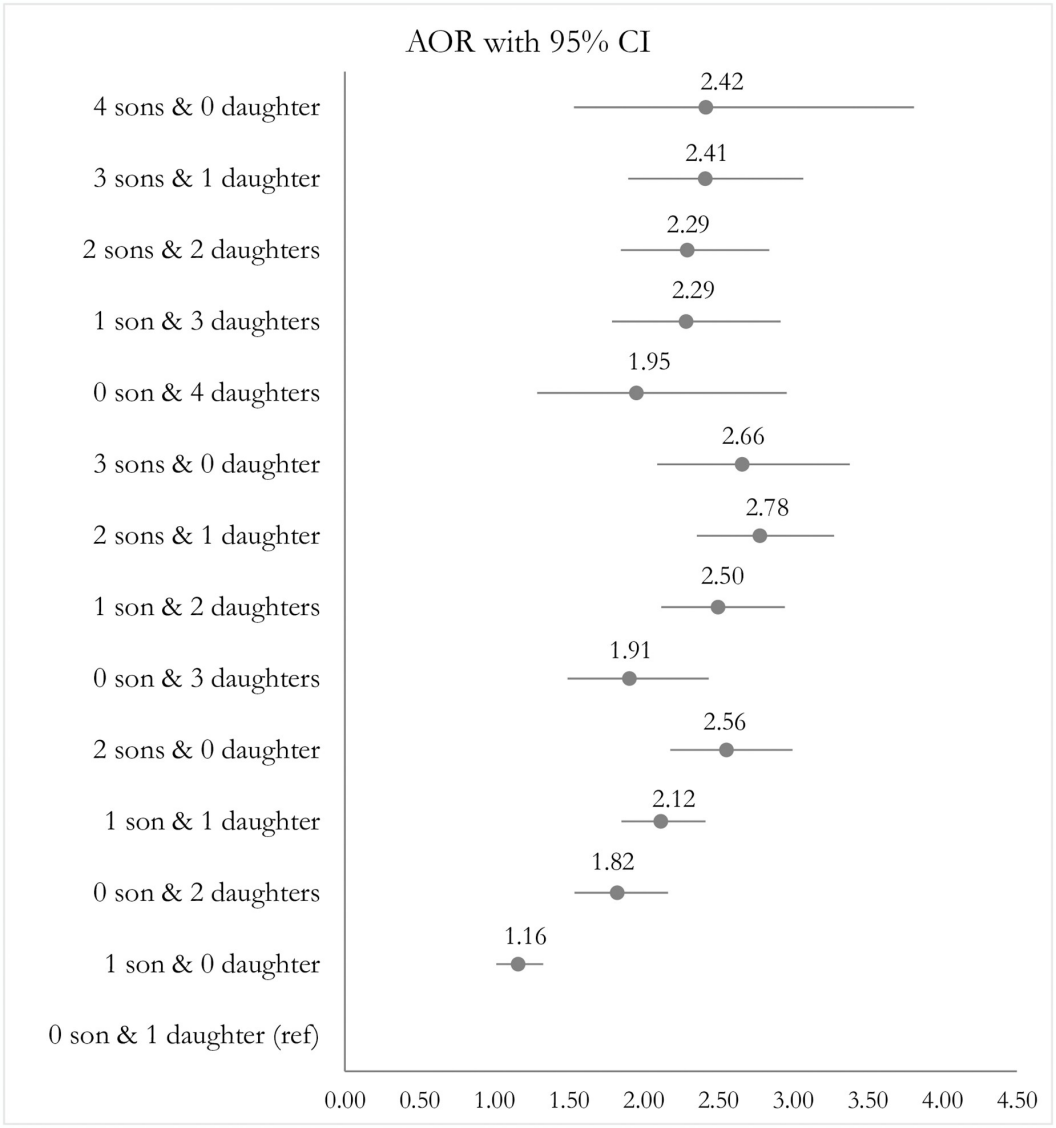
Effects of parity and sex composition on current use of modern contraceptive methods, controlling for covariates mentioned in Table 3.

When we examined both parity and children’s sex composition in a regression model, as presented in Fig 2 (the complete results of the relevant full model are provided in S3 Table), we found that, in each parity category, the aORs of using modern contraceptives tend to rise with an increasing number of sons. The aORs are significantly higher for all compositions of children’s sex when compared with the reference category of one daughter only. For instance, women with one son only had 16% (aOR, 1.16; 95% CI, 1.01–1.33) higher odds of reporting current use of modern contraception (Fig 2). Similarly, women with two daughters and no son, one son and one daughter, and two sons had 1.82, 2.12 and 2.56 times the odds of reporting the use of modern contraceptives, respectively, compared to women with only one daughter. Women with one son but no daughter were also less likely than women with at least one son and one daughter to report the current use of modern contraception.

## Discussion

The results suggest that the sex composition of children is a substantial factor in women’s current use of modern contraceptives. Women with only daughters, regardless of the number, were less likely to report currently using modern contraceptives compared with women who had one son and one daughter. These findings also indicate that women’s current use of modern contraceptive methods is also determined by the number of children. The desire to have both sons and daughters while keeping the family size relatively small could be a key reason for the stagnation and relatively low level of current use of modern contraception in Bangladesh.

The preference for at least one son is shaped by a complex interplay of social, cultural and economic factors. In Bangladesh and most other LMICs, sons are viewed as breadwinners with better job opportunities, leading to improved financial security for the family [25,28,29]. Additionally, patriarchal societies like Bangladesh grant men the majority of power and decision-making authority, with having a son seen as a way to secure a male heir to continue the family line and name [29]. Sons are also expected to be the primary caregivers for their parents in old age [25,30]. In contrast, daughters are often seen as weak and less capable of maintaining the family line due to moving to their husbands’ families following marriage, which is also perceived to limit their ability to provide care for their parents in old age [30].

The preference for a male child can lead couples to continue having children until they have at least one son, which can increase the size of their family and create a financial burden. This has a substantial impact on a densely populated country like Bangladesh. Also, adverse maternal and child health outcomes are more likely to occur with increased parity, including maternal anemia, stillbirth, preterm birth, low birth weight, pregnancy complications, as well as increased risks of maternal and child mortality [31–33]. Moreover, children from larger families are more likely to experience nutritional disorders such as stunting, wasting, and being underweight, which can impact their early childhood development [34] and educational performance [31,35–37]. These burdens are often greater for daughters, who may be undesired by their parents in families with higher parity [38,39]. As a result, parents may not prioritize their daughter’s education and instead focus on arranging their marriage at an early age, leading to intergenerational effects that increase the occurrence of adverse maternal and child health outcomes.

The intention to have a son also motivates some parents to abort their female fetuses [28]. This could be a reason for the imbalance in the sex ratio for children in the 2022 national population census [40]. Similar observations were reported in other South Asian countries [28]. Moreover, almost all abortions in Bangladesh occur outside of formal healthcare facilities by unskilled healthcare personnel because abortion is allowed only to save a woman’s life [41,42]. This practice further increases adverse maternal health outcomes, including post-abortion complications, which account for 13% of the total maternal deaths in Bangladesh [42]. Maternal healthcare service use is also found to be lower among women who experienced unintended pregnancies, as well as among women with higher parity [43].

In this study, we also found that women’s experiences of the death of one of their children, their increasing age, being in the richest wealth quintile, residing in rural areas and living in the Chattogram division region are associated with a lower likelihood of current use of modern contraception. While investigating the reasons for these associations is outside the scope of the current study, we can endeavour to justify them. Women’s experience of one of their children’s deaths may be linked to their fear of losing another child, which could make them less likely to use contraception with the intention of having more children [44]. The significantly lower prevalence of the current use of modern contraception by women living in rural areas and the Chattogram division could be due to a lack of knowledge of the importance of using contraception and limited access to family planning services [45]. The reasons for the negative effects of increasing age may be related to relatively low libido and sexual intercourse. However, the reason for women in the richest wealth quintile to report a lower level of current use of modern contraception than women in the poorest wealth quintiles remains unknown. Further research is needed to explore this issue.

The rising odds of using modern contraceptives with the number of living children can be attributed to women’s perception of achieving their desired family size or unwillingness to have a relatively large family [21,22,25]. This finding is consistent with that reported by Jayaraman et al [46]. Women’s growing knowledge about the risks of adverse maternal and child health outcomes, particularly for higher-order children, may also motivate them to use modern contraception [21], even if they do not have a male child. Javed and Mughal (2022), in their study using Pakistan DHS data, found that son preference was stronger at higher parities [47]. If we consider that not using modern contraceptives is to some extent attributed to their desire for a son, particularly among those with higher parities, our finding is not consistent with that of Javed and Mughal (2022). Rather, there may be tension in that while women prefer to have a son, they are also concerned about making the family large.

This study has several strengths and a few limitations. The main strength is the analysis of a large nationally representative dataset, which allowed adjustment for a range of factors at the individual-, household-, and community-levels. Moreover, the Poisson regression made our findings valid, precise and suitable for making national-level policies and programmes. However, this study is based on the analysis of cross-sectional survey data, which means that the associations reported are only correlational and not causal. Additionally, the information in the survey was recorded retrospectively with no scope for validation, which might have led to recall bias and reporting errors. However, any such errors are likely to be random. Furthermore, social and community-level norms play an important role in deciding the use and non-use of contraceptive methods, in addition to the factors considered in this study. It is important to adjust for these factors in the models, but we could not do so due to a lack of relevant data.

## Conclusion

Nearly 55% of women reported using modern contraceptive methods. Son preference may be a significant factor contributing to not using modern contraception. Women also want to have a mixed composition of sons and daughters. These desires could be a reason for the long-standing stagnation of modern contraceptive use in Bangladesh. We recommend the existing family planning programmes take social and cultural norms and preferences for male children into due consideration and intervene with tailored services to increase the uptake and regular use of modern contraception.

## Supporting information

S1 Table Association of use of modern contraceptives with the factors related to child, mother, household and community. (DOCX)

S2 Table Multilevel logistic regression examining the associations of women’s current use of modern contraceptives with child, mother, household and community characteristics (detailed model of Table 3 in the manuscript). (DOCX)

S3 Table Effects of parity and sex composition on current use of modern contraceptive methods, controlling for covariates mentioned in Table 3 (Detailed model of Fig 2 in the manuscript). (DOCX)

## References

[1] General Economic Division. Bangladesh Progress Report 2015. Dhaka, Bangladesh: Bangladesh Planning Commission, Governmen’s of the People Republic of Bangladeh, 2015.

[2] Khan MA, Khan N, Rahman O, Mustagir G, Hossain K, Islam R, et al. Trends and projections of under-5 mortality in Bangladesh including the effects of maternal high-risk fertility behaviours and use of healthcare services. PloS one. 2021;16(2):e0246210. doi: 10.1371/journal.pone.0246210 33539476 PMC7861360

[3] Khan M. Effects of unintended pregnancy on maternal healthcare services use in Bangladesh. PhD thesis, Faculty of Health and Medicine, School of Medicine and Public Health, The University of Newcastle, Australia. 2020.

[4] National Institute of Population Research and Training (NIPORT) et al. Bangladesh Demographic and Health Survey 2017–18. Dhaka, Bangladesh, and Rockville, Maryland, USA: NIPORT and ICF: 2020.

[5] Munir MMH. SDGS and Bangladesh: Progress, Challenges and Missing linkes. https://www.socialwatch.org/node/18286. Accessed June 22, 2022.

[6] Rahman MM. Role of proximate determinants in recent and past fertility stalls in Bangladesh. Biodemography Soc Biol. 2020;65(2):119–36. doi: 10.1080/19485565.2019.1683713 32432938

[7] Khan MN, Islam MM. Exploring rise of pregnancy in Bangladesh resulting from contraceptive failure. Sci Rep. 2022;12(1):2353. doi: 10.1038/s41598-022-06332-2 35149755 PMC8837649

[8] Pradhan J, Dwivedi R. Why unmet need for family planning remains high in Bangladesh: a community level analysis. Journal of Women’s Health Care. 2015;4(8):1–7. doi: 10.4172/2167-0420.1000290

[9] Pokhrel T, Aryal K, Adhikari R, Dulal BP, Karki DK, Dahal HR, et al. Socioeconomic Determinants of Inequalities in the Use of Modern Contraception among Currently Married Women. Journal Nepal Health Research Council. 2022;19(4):705–11. doi: 10.33314/jnhrc.v19i04.3738 35615826

[10] Nketiah-Amponsah E, Ampaw S, Twumasi Baffour P. Socioeconomic determinants of use and choice of modern contraceptive methods in Ghana. Tropopical Medical Health. 2022;50(1):33. doi: 10.1186/s41182-022-00424-5 35581604 PMC9116020

[11] Lasong J, Bougangue B, Nyarko Agyeman Y. Modern contraceptive use among women of reproductive age in Zimbabwe: analysis of 1999–2015 Zimbabwe Demographic Health Survey. Eur J Contracept Reprod Health Care. 2022;27(6):445–53. doi: 10.1080/13625187.2022.2107198 35959761

[12] Alawode OA, Okeke SR, Sah RK, Bolarinwa OA. Prevalence and determinants of intention to use modern contraceptives among grand-multiparous women in sub-Saharan Africa. Arch Public Health. 2022;80(1):246. doi: 10.1186/s13690-022-01006-x 36463217 PMC9719656

[13] Meselu W, Habtamu A, Woyraw W, Birlew Tsegaye T. Trends and predictors of modern contraceptive use among married women: Analysis of 2000–2016 Ethiopian Demographic and Health Surveys. Public Health Pract (Oxf). 2022;3:100243. doi: 10.1016/j.puhip.2022.100243 36101770 PMC9461593

[14] Boadu I. Coverage and determinants of modern contraceptive use in sub-Saharan Africa: further analysis of demographic and health surveys. Reproductive Health. 2022;19(1):18. doi: 10.1186/s12978-022-01332-x 35062968 PMC8781110

[15] Bellizzi S, Pichierri G, Menchini L, Barry J, Sotgiu G, Bassat Q. The impact of underuse of modern methods of contraception among adolescents with unintended pregnancies in 12 low- and middle-income countries. Journal of Global Health. 2019;9(2):020429. doi: 10.7189/jogh.09.020429 31673342 PMC6815657

[16] Khan MN, Akter S, Islam MM. Availability and readiness of healthcare facilities and their effects on long-acting modern contraceptive use in Bangladesh: analysis of linked data. BMC Health Service Research. 2022;22(1):1180. doi: 10.1186/s12913-022-08565-3 36131314 PMC9490900

[17] Islam MK, Haque MR, Hema PS. Regional variations of contraceptive use in Bangladesh: A disaggregate analysis by place of residence. PLoS One. 2020;15(3):e0230143. doi: 10.1371/journal.pone.0230143 32210443 PMC7094853

[18] Khan MN, Harris M, Loxton D. Modern Contraceptive Use Following an Unplanned Birth in Bangladesh: An Analysis of National Survey Data. International Perspectives on Sexual and Reproductive Health. doi: 10.1363/46e8820 32401729

[19] Schuler SR, Hashemi SM, Cullum A, Hassan M. The advent of family planning as a social norm in Bangladesh: women’s experiences. Reproductive Health Matters. 1996;4(7):66–78. https://www.jstor.org/stable/3775352.

[20] Eliason S, Baiden F, Tuoyire DA, Awusabo-Asare K. Sex composition of living children in a matrilineal inheritance system and its association with pregnancy intendedness and postpartum family planning intentions in rural Ghana. Reproductive health. 2018;15:1–10. doi: 10.1186/s12978-018-0616-2 30413219 PMC6234793

[21] Paudel YR, Acharya K. Fertility limiting intention and contraceptive use among currently married men in Nepal: evidence from Nepal demographic and health survey 2016. BioMed Research International. 2018;2018. doi: 10.1155/2018/5970705 30671463 PMC6323452

[22] Dey AK, Acharya R, Tomar S, Silverman JG, Raj A. How does the sex composition of children affect men’s higher ideal family size preference relative to women and contraceptive use patterns among couples? A cross-sectional analysis of dyadic couple’s data in India. SSM Popul Health. 2021;15:100835. doi: 10.1016/j.ssmph.2021.100835 34159248 PMC8193613

[23] Jayaraman A, Mishra V, Arnold F. The relationship of family size and composition to fertility desires, contraceptive adoption and method choice in South Asia. I International Perspectives on Sexual and Reproductive Health. 2009;35(1):29–38. doi: 10.1363/ifpp.35.029.09 19465346

[24] Robey B. Sex preference and fertility: what is the link? Asia Pac Pop Policy. 1987;(2):1–4. doi: 10.2307/2133408 12291498

[25] Asadullah MN, Mansoor N, Randazzo T, Wahhaj Z. Is son preference disappearing from Bangladesh? World Development. 2021;140:105353. doi: 10.1016/j.worlddev.2020.105353

[26] Polis C, Bradley SE, Bankole A, Onda T, Croft TN, Singh S. Contraceptive failure rates in the developing world: an analysis of demographic and health survey data in 43 countries. Guttmacher Institute, 2016. 10.1016/j.contraception.2016.03.011 PMC4970461 27018154

[27] Rahman M, Da Vanzo J. Gender preference and birth spacing in Matlab, Bangladesh. Demography. 1993;30(3):315–32. 8405601

[28] Calhoun LM, Nanda P, Speizer IS, Jain M. The effect of family sex composition on fertility desires and family planning behaviors in urban Uttar Pradesh, India. Reproductive Health. 2013;10:1–11. doi: 10.1186/1742-4755-10-48 24025670 PMC3848571

[29] Dey AK, Acharya R, Tomar S, Silverman JG, Raj A. How does the sex composition of children affect men’s higher ideal family size preference relative to women and contraceptive use patterns among couples? A cross-sectional analysis of dyadic couple’s data in India. SSM-Population Health. 2021;15:100835. doi: 10.1016/j.ssmph.2021.100835 34159248 PMC8193613

[30] Rajan S, Nanda P, Calhoun LM, Speizer IS. Sex composition and its impact on future childbearing: a longitudinal study from urban Uttar Pradesh. Reproductive Health. 2018;15(1):35. doi: 10.1186/s12978-018-0482-y 29486802 PMC5830318

[31] Garces A, Perez W, Harrison MS, Hwang KS, Nolen TL, Goldenberg RL, et al. Association of parity with birthweight and neonatal death in five sites: The Global Network’s Maternal Newborn Health Registry study. Reproductive Health. 2020;17(3):1–7. doi: 10.1186/s12978-020-01025-3 33334362 PMC7745358

[32] Lin L, Lu C, Chen W, Li C, Guo VY. Parity and the risks of adverse birth outcomes: a retrospective study among Chinese. BMC Pregnancy and Childbirth. 2021;21(1):1–11. doi: 10.1186/s12884-021-03718-4 33771125 PMC8004392

[33] Kozuki N, Lee AC, Silveira MF, Sania A, Vogel JP, Adair L, et al. The associations of parity and maternal age with small-for-gestational-age, preterm, and neonatal and infant mortality: a meta-analysis. BMC Public Health. 2013;13:1–10. doi: 10.1186/1471-2458-13-S3-S2 24564800 PMC3847520

[34] Islam MM, Khan MN. Early childhood development and its association with maternal parity. Child: Care, Health and Development. 2023;49(1):80–9. doi: 10.1111/cch.13011 35384014 PMC10084392

[35] Tesfaye A, Sisay G, Kabthymer RH, Tesfaye T. Under-nutrition and associated factors among pregnant women in public health care hospitals of Gedeo Zone, southern Ethiopia: A cross-sectional study. Heliyon. 2022;8(5):e09511. doi: 10.1016/j.heliyon.2022.e09511 35647358 PMC9136312

[36] Dekker LH, Mora-Plazas M, Marín C, Baylin A, Villamor E. Stunting associated with poor socioeconomic and maternal nutrition status and respiratory morbidity in Colombian school children. Food and nutrition bulletin. 2010;31(2):242–50. doi: 10.1177/156482651003100207 20707230

[37] Sari K, Sartika RAD. The effect of the physical factors of parents and children on stunting at birth among newborns in Indonesia. Journal of Preventive Medicine and Public Health. 2021;54(5):309. doi: 10.3961/jpmph.21.120 34649393 PMC8517371

[38] Thurstans S, Opondo C, Seal A, Wells JC, Khara T, Dolan C, et al. Understanding sex differences in childhood undernutrition: a narrative review. Nutrients. 2022;14(5):948. doi: 10.3390/nu14050948 35267923 PMC8912557

[39] Haq I, Hossain MI, Parvin MM, Saleheen AAS, Habib MJ, Chowdhury I-A-Q. Gender differences in child nutrition status of Bangladesh: a multinomial modeling approach. Journal of Humanities and Applied Social Sciences. 2022;4(5):379–92. doi: 10.1108/JHASS-02-2021-0030

[40] Bangladesh Bureau of Statistics. Population and Housing Census 2022: Preliminary Report Bangladesh Bureau of Statistics, Ministry of Planing, Dhaka, Bangladesh. 2022.

[41] Crouthamel B, Pearson E, Tilford S, Hurst S, Paul D, Aqtar F, et al. Out-of-clinic and self-managed abortion in Bangladesh: menstrual regulation provider perspectives. Reproductive Health. 2021;18(1):1–12. doi: 10.1186/s12978-021-01123-w 33766050 PMC7993471

[42] Hossain A, Maddow-Zimet I, Singh S, Remez L. Menstrual regulation, unsafe abortion and maternal health in Bangladesh. Guttmacher Institute, 2012. 23155545

[43] Khan MN, Harris ML, Shifti DM, Laar AS, Loxton D. Effects of unintended pregnancy on maternal healthcare services utilization in low-and lower-middle-income countries: systematic review and meta-analysis. International Journal of Public Health. 2019;64:743–54. doi: 10.1007/s00038-019-01238-9 31041453

[44] Mensch BS. The effect of child mortality on contraceptive use and fertility in Colombia, Costa Rica and Korea. Population Studies. 1985;39(2):309–27.

[45] Khan MN, Akter S, Islam MM. Availability and readiness of healthcare facilities and their effects on long-acting modern contraceptive use in Bangladesh: analysis of linked data. BMC Health Services Research. 2022;22(1):1180. doi: 10.1186/s12913-022-08565-3 36131314 PMC9490900

[46] Jayaraman A, Mishra V, Arnold F. The relationship of family size and composition to fertility desires, contraceptive adoption and method choice in South Asia. International Perspectives on Sexual and Reproductive Health. 2009:29–38. doi: 10.1363/ifpp.35.029.09 19465346

[47] Javed R, Mughal M. Changing patterns of son preference and fertility in Pakistan. Journal of International Development. 2022;34(6):1086–109. doi: 10.1002/jid.3618

