# Supplementary material for: Children’s sex composition and modern contraceptive use among mothers in Bangladesh
Source: PLoS One. 2024 May 31;19(5):e0297658. doi: 10.1371/journal.pone.0297658 (PMC11142447; doi:10.1371/journal.pone.0297658)
Supplement: S1 Table — (DOCX) [file pone.0297658.s001.docx]

**Table S1. Association of use of modern contraceptives with the factors related to child, mother, household and community**

| **Variables** | **Modern contraceptive use** | | **Total** | **P-values** |
| --- | --- | --- | --- | --- |
|  | **Not using** | **Using** |  |  |
| **Children characteristics** |  |  |  |  |
| **Parity** |  |  |  | p<0.001 |
| 1 | 1888 (23.92) | 1956 (20.73) | 3844 (22.18) |  |
| 2 | 2168 (27.47) | 3327 (35.25) | 5495 (31.7) |  |
| 3 | 1636 (20.73) | 2222 (23.54) | 3859 (22.26) |  |
| 4 | 1079 (13.67) | 1072 (11.36) | 2151 (12.41) |  |
| 5 or more | 1122 (14.21) | 861 (9.12) | 1983 (11.44) |  |
| **History of the death of children** |  |  |  | p<0.001 |
| None | 6348 (80.42) | 8077 (85.57) | 14425 (83.22) |  |
| At least one | 1545 (19.58) | 1362 (14.43) | 2907 (16.78) |  |
| **Sex composition of the existing children** |  |  |  | p<0.001 |
| No son | 1773 (22.46) | 1882 (19.94) | 3655 (21.09) |  |
| At least one son | 6121 (77.54) | 7557 (80.06) | 13678 (78.91) |  |
| ***Mother’s characteristics*** |  |  |  |  |
| **Age-groups** |  |  |  | p<0.001 |
| 15-19 | 357 (4.52) | 607 (6.43) | 964 (5.56) |  |
| 20-34 | 3454 (43.76) | 5640 (59.75) | 9094 (52.47) |  |
| ³35 | 4083 (51.72) | 3192 (33.82) | 7275 (41.97) |  |
| **Education level** |  |  |  | p<0.001 |
| No formal education | 1734 (21.97) | 1420 (15.05) | 3154 (18.2) |  |
| Primary | 2572 (32.58) | 3118 (33.04) | 5690 (32.83) |  |
| Secondary | 2815 (35.66) | 3833 (40.61) | 6648 (38.36) |  |
| Higher | 773 (9.79) | 1067 (11.31) | 1840 (10.61) |  |
| **Respondent's employment status** |  |  |  | p<0.001 |
| Unemployed | 4153 (52.61) | 4473 (47.39) | 8626 (49.77) |  |
| Employed | 3741 (47.39) | 4965 (52.61) | 8706 (50.23) |  |
| **Religion** |  |  |  | p<0.001 |
| Muslim | 7245 (91.79) | 8414 (89.14) | 15659 (90.34) |  |
| Others | 648 (8.21) | 1025 (10.86) | 1673 (9.66) |  |
| **Exposure to mass media** |  |  |  | p<0.001 |
| Low | 2900 (36.73) | 3120 (33.05) | 6020 (34.73) |  |
| Moderate | 4340 (54.98) | 5447 (57.7) | 9787 (56.46) |  |
| High | 654 (8.28) | 872 (9.24) | 1526 (8.81) |  |
| ***Household’s characteristics*** |  |  |  |  |
| **Husband’s education** |  |  |  | p<0.01 |
| No formal education | 1607 (23.36) | 2179 (23.11) | 3786 (23.22) |  |
| Primary | 2113 (30.71) | 3200 (33.95) | 5313 (32.58) |  |
| Secondary | 2096 (30.46) | 2643 (28.03) | 4738 (29.06) |  |
| Higher | 1064 (15.46) | 1406 (14.91) | 2470 (15.14) |  |
| **Husband’s occupation** |  |  |  | p<0.001 |
| Agriculture | 1737 (26.22) | 2706 (29.09) | 4443 (27.9) |  |
| Labourer | 3321 (50.16) | 3927 (42.22) | 7248 (45.52) |  |
| Services | 335 (5.05) | 485 (5.22) | 820 (5.15) |  |
| Business | 1230 (18.57) | 2183 (23.47) | 3413 (21.43) |  |
| **Wealth index** |  |  |  | p<0.001 |
| Poorest | 1356 (17.18) | 1941 (20.56) | 3297 (19.02) |  |
| Poorer | 1559 (19.76) | 1884 (19.96) | 3443 (19.87) |  |
| Middle | 1660 (21.03) | 1869 (19.81) | 3529 (20.36) |  |
| Rich | 1600 (20.28) | 1932 (20.47) | 3532 (20.38) |  |
| Richest | 1717 (21.75) | 1812 (19.2) | 3529 (20.36) |  |
| ***Community-level characteristics*** |  |  |  |  |
| **Place of residence** |  |  |  | p<0.01 |
| Rural | 2095 (26.54) | 2788 (29.53) | 4882 (28.17) |  |
| Urban | 5799 (73.46) | 6651 (70.47) | 12450 (71.83) |  |
| **Region** |  |  |  | p<0.001 |
| Barishal | 471 (5.97) | 512 (5.43) | 983 (5.67) |  |
| Chattogram | 1641 (20.79) | 1495 (15.83) | 3135 (18.09) |  |
| Dhaka | 1895 (24) | 2410 (25.53) | 4304 (24.83) |  |
| Khulna | 945 (11.97) | 1089 (11.54) | 2034 (11.74) |  |
| Mymensingh | 543 (6.87) | 774 (8.2) | 1317 (7.6) |  |
| Rajshahi | 1045 (13.24) | 1400 (14.84) | 2445 (14.11) |  |
| Rangpur | 825 (10.45) | 1283 (13.59) | 2108 (12.16) |  |
| Sylhet | 530 (6.71) | 475 (5.04) | 1005 (5.8) |  |
