# Supplementary material for: Children’s sex composition and modern contraceptive use among mothers in Bangladesh
Source: PLoS One. 2024 May 31;19(5):e0297658. doi: 10.1371/journal.pone.0297658 (PMC11142447; doi:10.1371/journal.pone.0297658)
Supplement: S2 Table — (DOCX) [file pone.0297658.s002.docx]

**Table S2. Multilevel logistic regression examining the associations of women’s current use of modern contraceptives with child, mother, household and community characteristics (detailed model of Table 3 in the manuscript)**

Number of observations: 15,836

Number of groups: 672

Log likelihood: -10062.246

Wald chi-2: 1051.48; p<0.0001

| **Variables** | | | **Women currently using modern contraception methods** | | | | |
| --- | --- | --- | --- | --- | --- | --- | --- |
|  | | |  | | | **95% CI** | |
| **Parity** | | | **aOR** | **SE** | **P>z** | **Lower limit** | **Upper limit** |
| 1 (reference) | | | 1 |  |  |  |  |
| 2 | | | 1.83 | 0.10 | 0.000 | 1.65 | 2.04 |
| 3 | | | 2.17 | 0.14 | 0.000 | 1.92 | 2.46 |
| 4 | | | 1.99 | 0.15 | 0.000 | 1.71 | 2.31 |
| 5 or more | | | 1.91 | 0.16 | 0.000 | 1.61 | 2.27 |
| **History of the death of a child women gave birth to** | | |  |  |  |  |  |
| None (reference) | | | 1 |  |  |  |  |
| At least one | | | 0.76 | 0.04 | 0.000 | 0.69 | 0.85 |
| **Sex composition of the existing children** | | |  |  |  |  |  |
| No son (reference) | | | 1 |  |  |  |  |
| At least one son | | | 1.22 | 0.06 | 0.000 | 1.12 | 1.34 |
| ***Mother's characteristics*** | | |  |  |  |  |  |
| **Age-group** | | |  |  |  |  |  |
| 15-19 (reference) | | | 1 |  |  |  |  |
| 20-34 | | | 0.59 | 0.05 | 0.000 | 0.50 | 0.69 |
| ≥35 | | | 0.25 | 0.02 | 0.000 | 0.20 | 0.29 |
| **Education level** | | |  |  |  |  |  |
| Not formal education (reference) | | | 1 |  |  |  |  |
| Primary | | | 1.23 | 0.07 | 0.000 | 1.10 | 1.37 |
| Secondary | | | 1.40 | 0.09 | 0.000 | 1.24 | 1.58 |
| Higher | | | 1.66 | 0.15 | 0.000 | 1.39 | 1.98 |
| **Respondent’s employment status** | | |  |  |  |  |  |
| Unemployed (reference) | | | 1 |  |  |  |  |
| Employed | | | 1.31 | 0.05 | 0.000 | 1.21 | 1.41 |
| ***Household’s characteristics*** | | |  |  |  |  |  |
| **Husband’s education** | | |  |  |  |  |  |
| No formal education (reference) | | | 1 |  |  |  |  |
| Primary | | | 0.96 | 0.05 | 0.255 | 0.87 | 1.06 |
| Secondary | | | 0.79 | 0.05 | 0.000 | 0.71 | 0.89 |
| Higher | | | 0.76 | 0.06 | 0.000 | 0.65 | 0.89 |
| **Husband’s occupation** | | |  |  |  |  |  |
| Agriculture (reference) | | | 1 |  |  |  |  |
| Physical | | | 0.66 | 0.03 | 0.000 | 0.60 | 0.72 |
| Services | | | 0.99 | 0.09 | 0.538 | 0.82 | 1.21 |
| Business | | | 1.10 | 0.06 | 0.294 | 0.98 | 1.22 |
| **Wealth index** | | |  |  |  |  |  |
| Poorest (reference) | | | 1 |  |  |  |  |
| Poorer | | | 0.76 | 0.05 | 0.001 | 0.68 | 0.85 |
| Middle | | | 0.70 | 0.05 | 0.000 | 0.62 | 0.80 |
| Rich | | | 0.66 | 0.05 | 0.000 | 0.58 | 0.76 |
| Richest | | | 0.51 | 0.05 | 0.000 | 0.44 | 0.60 |
| **Religion** | | |  |  |  |  |  |
| Muslim (reference) | | | 1 |  |  |  |  |
| Others | | | 1.34 | 0.09 | 0.000 | 1.17 | 1.53 |
| **Exposure to mass media** | | |  |  |  |  |  |
| No (reference) | | | 1 |  |  |  |  |
| Moderate | | | 1.27 | 0.06 | 0.000 | 1.16 | 1.38 |
| High | | | 1.36 | 0.10 | 0.000 | 1.17 | 1.59 |
| ***Community-level characteristics*** | | |  |  |  |  |  |
| **Place of residence** | | |  |  |  |  |  |
| Rural (reference) | | | 1 |  |  |  |  |
| Urban | | | 1.53 | 0.03 | 0.000 | 1.36 | 1.72 |
| **Regions** | | |  |  |  |  |  |
| Barishal (reference) | | | 1 |  |  |  |  |
| Chattogram | | | 0.83 | 0.08 | 0.048 | 0.68 | 1.03 |
| Dhaka | | | 1.20 | 0.11 | 0.057 | 0.98 | 1.48 |
| Khulna | | | 0.98 | 0.09 | 0.748 | 0.79 | 1.22 |
| Mymensingh | | | 1.21 | 0.12 | 0.027 | 0.96 | 1.52 |
| Rajshahi | | | 1.22 | 0.11 | 0.039 | 0.98 | 1.51 |
| Rangpur | | | 1.21 | 0.11 | 0.090 | 0.97 | 1.50 |
| Sylhet | | | 0.86 | 0.08 | 0.108 | 0.68 | 1.10 |
| **Constant** | | | 1.61 | 0.32 | 0.000 | 1.23 | 2.11 |
|  | | |  |  |  |  |  |
| ***p* for LR test vs. logistic regression** | | | 185.5 |  |  |  |  |
| **Random effect variance** | | | 1.60 | 0.03 |  | 0.13 | 0.20 |
|  |  |  | | | | | |

Note: Survey weight was applied in the regression
