# Supplementary material for: Children’s sex composition and modern contraceptive use among mothers in Bangladesh
Source: PLoS One. 2024 May 31;19(5):e0297658. doi: 10.1371/journal.pone.0297658 (PMC11142447; doi:10.1371/journal.pone.0297658)
Supplement: S3 Table — (DOCX) [file pone.0297658.s003.docx]

**Table S3. Effects of parity and sex composition on current use of modern contraceptive methods, controlling for covariates mentioned in Table 3 (Detailed model of Figure 2 in the manuscript)**

Number of observations: 14,705

Number of groups: 672

Log likelihood: -9274.7468

Wald chi-2: 988.69; p<0.0001

| **Variables** | **AOR** | **SE** | **P>z** | **95% Confidence Interval** | |
| --- | --- | --- | --- | --- | --- |
|  |  |  |  | **Lower limit** | **Upper limit** |
| **Sibling sex composition** |  |  |  |  |  |
| 0 son & 1 daughter | 1.00 |  |  |  |  |
| 1 son & 0 daughter | 1.16 | 0.08 | 0.030 | 1.01 | 1.33 |
| 0 son & 2 daughters | 1.82 | 0.16 | 0.000 | 1.54 | 2.16 |
| 1 son & 1 daughter | 2.12 | 0.14 | 0.000 | 1.85 | 2.42 |
| 2 sons & 0 daughter | 2.56 | 0.21 | 0.000 | 2.18 | 3.00 |
| 0 son & 3 daughters | 1.91 | 0.24 | 0.000 | 1.49 | 2.44 |
| 1 son & 2 daughters | 2.50 | 0.21 | 0.000 | 2.12 | 2.95 |
| 2 sons & 1 daughter | 2.78 | 0.23 | 0.000 | 2.36 | 3.28 |
| 3 sons & 0 daughter | 2.66 | 0.33 | 0.000 | 2.09 | 3.38 |
| 0 son & 4 daughters | 1.95 | 0.41 | 0.002 | 1.29 | 2.96 |
| 1 son & 3 daughters | 2.29 | 0.29 | 0.000 | 1.79 | 2.92 |
| 2 sons & 2 daughters | 2.29 | 0.25 | 0.000 | 1.85 | 2.84 |
| 3 sons & 1 daughter | 2.41 | 0.30 | 0.000 | 1.90 | 3.07 |
| 4 sons & 0 daughter | 2.42 | 0.56 | 0.000 | 1.53 | 3.81 |
| **Older children died** |  |  |  |  |  |
| None | 1.00 |  |  |  |  |
| 1. At least one | 0.85 | 0.04 | 0.001 | 0.76 | 0.94 |
| **Age-groups** |  |  |  |  |  |
| 15-19 | 1.00 |  |  |  |  |
| 20-34 | 0.57 | 0.05 | 0.000 | 0.49 | 0.68 |
| 35+ | 0.22 | 0.02 | 0.000 | 0.18 | 0.27 |
| **Education level** |  |  |  |  |  |
| Not formal education | 1.00 |  |  |  |  |
| Primary | 1.33 | 0.08 | 0.000 | 1.18 | 1.50 |
| Secondary | 1.41 | 0.09 | 0.000 | 1.23 | 1.61 |
| Higher | 1.61 | 0.15 | 0.000 | 1.34 | 1.94 |
| **Respondents’ working status** |  |  |  |  |  |
| No | 1.00 |  |  |  |  |
| Yes | 1.26 | 0.05 | 0.000 | 1.17 | 1.37 |
| **Husband’s education** |  |  |  |  |  |
| No formal education | 1.00 |  |  |  |  |
| Primary | 0.98 | 0.05 | 0.652 | 0.88 | 1.09 |
| Secondary | 0.82 | 0.05 | 0.001 | 0.73 | 0.93 |
| Higher | 0.78 | 0.06 | 0.002 | 0.66 | 0.91 |
| **Husband’s occupation** |  |  |  |  |  |
| Agriculture | 1.00 |  |  |  |  |
| Physical | 0.65 | 0.03 | 0.000 | 0.59 | 0.72 |
| Services | 0.91 | 0.09 | 0.355 | 0.75 | 1.11 |
| Business | 1.04 | 0.06 | 0.517 | 0.93 | 1.17 |
| **Wealth Index** |  |  |  |  |  |
| Poorest | 1.00 |  |  |  |  |
| Poorer | 0.81 | 0.05 | 0.001 | 0.72 | 0.92 |
| Middle | 0.75 | 0.05 | 0.000 | 0.65 | 0.85 |
| Rich | 0.72 | 0.05 | 0.000 | 0.62 | 0.82 |
| Richest | 0.58 | 0.05 | 0.000 | 0.49 | 0.69 |
| **Religion** |  |  |  |  |  |
| Muslim | 1.00 |  |  |  |  |
| Others | 1.37 | 0.09 | 0.000 | 1.21 | 1.57 |
| **Exposure to mass media** |  |  |  |  |  |
| No | 1.00 |  |  |  |  |
| Moderate | 1.23 | 0.06 | 0.000 | 1.13 | 1.35 |
| High | 1.30 | 0.10 | 0.001 | 1.12 | 1.52 |
| **Place of residence** |  |  |  |  |  |
| Urban | 1.00 |  |  |  |  |
| Rural | 0.65 | 0.04 | 0.000 | 0.58 | 0.72 |
| **Regions** |  |  |  |  |  |
| Barishal | 1.00 |  |  |  |  |
| Chattogram | 0.79 | 0.08 | 0.014 | 0.66 | 0.95 |
| Dhaka | 1.12 | 0.11 | 0.222 | 0.93 | 1.35 |
| Khulna | 0.95 | 0.09 | 0.595 | 0.79 | 1.15 |
| Mymensingh | 1.23 | 0.12 | 0.040 | 1.01 | 1.49 |
| Rajshahi | 1.18 | 0.11 | 0.080 | 0.98 | 1.43 |
| Rangpur | 1.18 | 0.11 | 0.096 | 0.97 | 1.42 |
| Sylhet | 0.79 | 0.08 | 0.023 | 0.65 | 0.97 |
|  |  |  |  |  |  |
| Constant | 2.62 | 0.37 | 0.000 | 1.99 | 3.46 |
|  |  |  |  |  |  |
| Random effect parameters: Clusters |  |  |  |  |  |
| sd(Constant) | 0.37 | 0.03 |  | 0.32 | 0.43 |
